# Experiences and perceptions of birth companions supporting women in labour at a District Hospital in Limpopo, South Africa

**DOI:** 10.4102/curationis.v44i1.2186

**Published:** 2021-10-27

**Authors:** Joy V. Summerton, Tsakani R. Mtileni, Maphei E. Moshabela

**Affiliations:** 1 Department of Obstetrics and Gynaecology, Faculty of Health Sciences, University of Limpopo, Polokwane, South Africa

**Keywords:** birth companion, compassionate care, respectful care, childbirth, experience of care, Limpopo, maternal care

## Abstract

**Background:**

South Africa has included birth companions in its national guidelines for maternity care and the revised Maternity Case Record, in and effort to improve the quality and experience of care. However, reservations amongst healthcare providers remain about the acceptability of birth companions in the labour ward.

**Objectives:**

To document the experiences and perceptions of birth companions who supported women in labour in a rural hospital in Limpopo Province where a Respectful Maternity Care (RMC) project was piloted.

**Method:**

An institution-based cross-sectional study design was employed. Purposive sampling was employed where all birth companions who supported a woman during labour and birth were included in the study. The experiences and perceptions of birth companions were captured using a birth companion feedback book during the period of 1st April to 30th August 2019. Thematic analysis was used to analyse the data.

**Results:**

Seventy-one (71) of the 73 birth companions only had positive responses about the birthing experience and how both the birth companion and woman in labour were treated. Two birth companions were dissatisfied with the treatment provided by the midwife that supported the birth.

**Conclusion:**

It is important for healthcare providers to understand the far reaching emotional and psychological impact of their attitudes and behaviour on, not only women in labour but also on others who witness their (healthcare providers) behaviour. Mechanisms to obtain feedback from birth companions should be integrated into strategies to improve the quality and experience of care for women during childbirth.

## Introduction

Childbirth is a highly stressful event for many women, which requires the constant involvement of the mind and body, and evoking a wide range of positive and negative emotions (Carlson & Pettersson 2009; Story et al. 2012). Because of fear and anxiety, women in labour need continuous support and care from the healthcare providers in the labour ward. There is a heath plethora of evidence to support the benefits of continuous support of women during labour and birth. A number of World Health Organization (WHO) guidelines recommend continuous support by a companion of choice during labour and childbirth (WHO 2012, 2014, 2015, 2016a, 2016b, 2018). A Cochrane review of continuous support during labour and childbirth revealed that women who received continuous support were more likely to have shorter labour, experience improved satisfaction with care and have spontaneous unassisted (no use of forceps or vacuum) vaginal delivery, thus reducing the need for caesarian sections. It also reduced the need for pain medication during labour, and their babies were less likely to have low (less than 7) 5-min Apgar scores (Afulani et al. 2018; Bohren et al. 2017; Hodnett et al. 2013; Kabakian-Khasholian & Portela 2017).

A birth companion is any person, chosen by a woman, to provide her with continuous support during labour and childbirth. The roles and responsibilities of a birth companion include providing emotional support (continuous reassurance), information about labour progress and advice regarding coping techniques, comfort measures (comforting touch, massages, promoting adequate fluid intake and output) and advocacy (helping the woman articulate her wishes to the healthcare provider team) (WHO 2016).

Despite the overwhelming evidence of the benefits of birth companionship, its implementation is not universal. A study in Brazil found that in spite of the legal obligation to provide a companion to support women during labour, birth companions are still not fully accepted in public hospitals (Souza & Gualda 2016). A study in Bangladesh revealed that women who gave birth in a health facility were less likely to be supported by a birth companion during labour and birth, the main reason cited being their lack of knowledge about the benefits of having a birth companion (Perkins et al. 2019). One other study in Limpopo found that none of the midwives encouraged the presence of a birth companion during labour and childbirth. This may be attributed to the negative attitude of the staff towards the presence of outsiders in the labour ward (Maputle 2018).

Providing continuous support to women during the stressful and anxiety-provoking time of labour and birth is an important responsibility of healthcare providers. Because of staff shortages, healthcare providers in many settings have limited time to provide continuous supportive care to women in labour. A study in Thailand found that many women were left to labour without being provided continuous support because of staff shortage (Yuenyong, Jirapaet & O’Brein 2008). Whilst another study in Limpopo, South Africa, reported that midwives were unable to provide individual care to women in labour as they were caring for more than one woman at the same time (Maputle 2018).

Research has unequivocally shown that women in labour have a profound need for continuous physical and emotional support from a companion of choice throughout labour and birth. This support is an integral part of the sensitive and responsive women-centred care, which improves women’s and newborn’s health outcomes (Ojelade et al. 2017).

A Respectful Maternity Care (RMC) project was piloted in a sub-district of Limpopo Province, South Africa. One of the key components of the RMC project was for pregnant women to identify a birth companion of choice to support them during pregnancy, labour, birth and postnatal period. The labour ward staff were orientated on the RMC approach and the role of birth companions during the pregnancy, labour and postnatal period. They were also instructed to adjust their infrastructure to accommodate birth companions by ensuring all labour rooms to have a chair and at least a curtain to maintain the privacy of the woman in labour. They were also equipped with information to capacitate birth companions (including pamphlets and posters) and supplied with birth companion toolkits, which comprised items to promote non-pharmacological pain relief, such as birthing balls, hand massages, massage cream, lip balm and so on.

A fair amount of resistance was experienced from frontline healthcare workers and managers during the project inception phase. Some of the impediments to successfully implement birth companionship that was identified by frontline healthcare workers and managers during buy-in and orientation workshops included a perceived reluctance of community members to accompany pregnant women into the labour ward and witness the birthing process, inadequate space for birth companions in the labour ward and compromising of client privacy in the presence of birth companions in the labour ward. A review of studies conducted in middle and lower-income countries revealed that in all the studies, healthcare providers displayed negative attitude towards birth companionship, citing the following reasons: fear of cross-infection and overcrowding in labour wards; the complexity of expected collaboration amongst women, their companions and healthcare providers and interference of birth companions with the clinical duties of healthcare providers (Afulani et al. 2018).

The purpose of this study was to ascertain and document the experiences of birth companions who support pregnant women during labour and childbirth, with a view to enhance insights on and advocate for the integration of birth companions of choice within the labour ward of the hospital where an RMC project was piloted.

## Research methods and design

### Study area

The study was conducted in a public sector district hospital, which is a referral hospital for 21 feeder primary healthcare facilities, and it contributes to more than 90% of facility deliveries/births in its catchment area (sub-district). The sub-district is constituted by 128 villages and one small industrial town, which makes up a total population of 212 701. The hospital has four First Stage of labour rooms, three Active Stage of labour rooms and one theatre for caesarian sections in the Maternity Ward. The hospital conducts between 400 and 500 births per month. The hospital was selected for the purpose of piloting an RMC project aimed at improving the quality and experience of care for pregnant women during labour and childbirth.

### Study design and period

An institution-based cross-sectional study design was employed. The experiences and perceptions of birth companions who supported women during labour and childbirth were captured using a birth companion feedback book during the period of 01 April 2019 to 30 August 2019.

### Population

Birth companions who supported women during labour and childbirth at the district hospital.

### Sample size and procedure

Purposive sampling was employed where all birth companions who supported a woman during labour and birth were expected to respond and thus included in the study. The midwife supporting each birth was tasked with encouraging each birth companion, who were present during labour and birth to share their experience of supporting a woman during childbirth.

Birth companions were encouraged, but not forced, to participate in the study. The purpose of the study was explained to all participants. They were also told that the information obtained from them would be used to improve the quality of care for pregnant women during labour and birth. They were given the option to remain anonymous and were assured that no harm or discriminatory action would befall them or the pregnant woman that they were supporting through participation or non-participation in the study. Birth companions providing feedback in the birth companion book after the purpose of the study was explained to them, was an implication of providing consent to participate in the study.

#### Operational definition

*Birth companion* is an adult male or female, selected by a pregnant woman, who provides her with continuous physical and emotional support throughout labour and birth.

### Data collection tool and procedure

A birth companion feedback book was developed and placed at the nurses’ station for all the birth companions to provide written feedback on their experience and perceptions whilst supporting a woman in labour and childbirth. The book has fields to fill in details such as the name of the woman in labour (optional), the birth companion’s name (optional), the relationship of the birth companion to the woman in labour, date of delivery, name of the midwife who supported the birth and feedback from the birth companion. Healthcare providers were tasked with encouraging the birth companions to document their experiences as soon after the birth as possible and before the woman is transferred from the labour ward to the postnatal ward. The birth companions had the option to record their feedback in their language of choice. The birth companion book was placed on the counter at the nurses’ station in the labour ward. Data from 01 April 2019 to 31 August 2019 were used for the study.

### Data processing and analysis

Copies of the data from the Birth Companion Feedback Book were made, as the book could not be removed from the nurses’ station where it was accessible to birth companions. The responses were read by two researchers to make sure that all responses were captured accurately, especially those that were illegible. The responses in *Sepedi* (local vernacular) were translated into English. Thematic analysis was used to analyse the data. Themes were identified, and then the data were coded and systematically placed in the identified themes. Similarities and contradictions were highlighted.

### Ethical considerations

Ethical approval was obtained from the University of Limpopoi Turfloop Research Ethics Committee (TREC/59/2019:IR).

## Results

The district hospital had 1860 births during the study period. Seventy-three (73) birth companions provided written feedback in the Birth Companion Feedback Book during the period between April to August 2019. The low response rate can have multiple interpretations, namely that the majority of mothers during the study period did not have a birth companion to support them during labour and birth (high probability as the concept of birth companionship was new to the community and facility) or not all birth companions documented the birthing experience in the birth companion book (also highly probable as the onus was on the midwives to inform birth companions about the book and encourage them to provide feedback). Seventy-one (71) of the 73 birth companions gave only positive responses about the birthing experience and how both the birth companion and woman in labour were treated. Two birth companions were dissatisfied with the treatment of the midwife that supported the delivery. The findings of the study are not generalisable in terms of the proportion of birth companions that had a positive experience versus the proportion of birth companions that had a negative experience during labour and birth. The findings do, however, provide valuable insights into the community acceptability of providing continuous physical and emotional support to women during labour and birth through the use of birth companions.

The majority (*n* = 71) of the birth companions were females, and most (58) were a relative of the birthing woman (mother, aunt, sister, grandmother, husband/partner), whilst the rest were volunteer birth companions with no prior relationship with the woman being supported.

The responses of the birth companions included appreciation for the perceived competency of the healthcare staff, as well as the compassionate and respectful care provided to the woman in labour by the staff. They also commended the staff for their positive attitudes towards the birth companions themselves.

### Types of support provided by a birth companion

Birth companions generally provide emotional and physical support to a woman in labour. This includes encouraging and supporting the woman in labour to be mobile (walking around), helping her to focus on the purpose of labour and its ultimate goal when she feels that she is unable to continue to the end of the birthing process, serving as an intermediary between the woman in labour and the healthcare team by helping the healthcare team to communicate effectively with the woman in labour who may find it difficult to interpret and synthesise information during intense contractions and high levels of anxiety and fear. Birth companions also help to effectively communicate the feelings, needs and desires of the woman in labour to the healthcare team when needed and facilitate non-pharmacological pain relief (Bohren et al. 2017; Hodnett et al. 2013; Ojelade et al. 2017; Yuenyong et al. 2008).

The physical and emotional support provided by birth companions is best articulated in the following responses of two volunteer birth companions who met the women in labour for the first time in the labour ward upon their admission:

‘She was booked for a c-section [*caesarian section*] because her labour was not progressing. After I encouraged her to walk around with me instead of lying on the bed the whole time, and we used the ball for her to sit on and bounce on … next thing the baby came and the c/section [*caesarian section*] was cancelled.’ (Participant 1, female, volunteer birth companion)

‘It was a pleasure being a birth companion to Y. She was very anxious and felt that she was not able to handle the pain and she kept begging for a c/section [*caesarian section*]. I lay on the floor with her, which is where she preferred to be, and I encouraged and supported her to use different breathing techniques as well as massaging her back and hands and reassuring her. She eventually focused on the breathing and my encouraging messages, which calmed her down and she stopped asking for a c/section. Congratulations to Y on the natural birth of her 2nd baby boy.’ (Participant 57, female, volunteer birth companion)

The perceived value and acceptability of a birth companion by healthcare providers are increased by the level of competency of a birth companion. One of the reasons cited by healthcare providers who are reluctant or resistant to integrating birth companions in the labour ward is that birth companions do not understand their role and thus become a hindrance to the healthcare team performing their clinical responsibilities. Ideally, birth companions should attend antenatal education classes in order to be equipped with the necessary knowledge and skills to provide effective physical and emotional support to the woman in labour and function as an integral member of the healthcare team supporting the woman in labour (Ojelade et al. 2017). The RMC pilot required all birth companions, upon arrival at the labour ward, to be orientated on what is expected of them, and the use of various non-pharmacological pain relief techniques. This contributed to the positive experience of birth companions who felt to be useful and part of the support team, as alluded to by the following respondents:

‘I feel like I was an angel for the mother. There were so many other mothers in the ward that were giving birth and the nurses were running around trying to attend to everyone. The mother that I was supporting was walking around the labour room with me and suddenly walked towards the door saying she needs to use the toilet urgently. Something inside me, and her facial expression and voice, told me to quickly go to one of the nurses to inform them. The nurse was busy helping another mother whose baby was already being born, and told me to ask the mother that I was supporting not to go to the toilet and she would attend to her very soon. We soon realised that the head was crowning and that is why the mother had the urge to go to the toilet. Luckily I was there to ask her not to go to the toilet and helped her onto the bed till the nurse arrived to help her deliver her baby. I am glad I was there for mom and her baby.’ (Participant 1, female, volunteer birth companion)

‘I felt blessed being with my aunt when she gave birth but I was scared a bit. It was great fun to help with everything. I would like to thank all the staff. They were all great. Big up to staff in Ward F.’ (Participant 46, female, birth companion of choice)

‘I was her birth companion since 07H00. I was encouraging her to stay strong and hang in there because when a woman is in labour, she must not give up because of the pain, but persevere till the end. I must encourage her and not be impatient with her. I thank the nurses and doctors at Hospital X for allowing me to be with her. Continue doing what you are doing.’ (Participant 39, female, birth companion of choice)

‘I felt happy to see my child give birth. I saw the whole process. From the first pain, until birth and even helping to massage. I give thanks to all the sisters who were responsible for the birth. Thank you.’ (Participant 62, female, birth companion of choice)

The RMC initiative advocates for women and family-centred care, which is grounded in compassion and respect for the needs of women in labour. Studies have shown that women in labour expect healthcare workers to demonstrate respect, empathy and personalised attention towards them, as opposed to neglect, rudeness, dismissive attitude and viewing them as merely another woman in labour in his or her shift (Yuenyong et al. 2008). The birth companions in the study bore testament to the kind and compassionate behaviour demonstrated by healthcare providers towards not only the women in labour but also to them as birth companions:

‘The service was good. They assisted us with love and respect and they used terms and regulations of the hospital.’ (Participant 24, female, birth companion of choice)

‘I would like to thank all the sisters who assisted my child to give birth. They were patient enough and they did a marvellous job. May God bless them to continue doing this to all patients.’ (Participant 33, female, birth companion of choice)

### Male partners as birth companions

Encouraging male partners to be birth companions is a contentious area, underpinned by cultural and practical considerations. In Thailand, men feel uncomfortable, unconfident and incompetent to meet either the physical or emotional needs of their partners in labour (Ledenfors & Bertero 2016). A consideration raised by healthcare providers in the RMC pilot was the acceptability of male companions by other women in labour in overcrowded maternity units. In spite of the reservations, the very few male birth companions in the study reported positive experiences:

‘I am very happy that I was able to accompany my partner into theatre so that I could hold her hand and allay her anxiety. She didn’t have to be there all alone. It was also very special for me to receive my baby and cut the umbilical cord.’ (Participant 6, male, birth companion of choice)

‘I am thankful to Hospital X for the good treatment that you gave my wife so that she could safely give birth to my first child.’ (Participant 36, male, birth companion of choice)

One male birth companion saw that his wife, who was in a critical condition, was further experiencing severe pregnancy-induced hypertension (PIH) when admitted in the labour ward. He was grateful for being allowed to remain at his wife’s side and hold her hand whilst awaiting her referral to a higher level of care (referral hospital). He was thankful to the staff for giving him the opportunity to play a reassuring and calming role to his wife, in spite of his own fears and anxiety:

‘I didn’t understand why this happened to my wife. Was it my fault? Did I put her in this situation? I had so many questions that I was asking the doctors and nurses but they didn’t have answers for me. I didn’t know what to do but they told me to just keep calm for her sake and I must just reassure her and let her know that I am there. So I just held her hand and spoke to her until the ambulance arrived to take her to Hospital X. I wish I could have taken her pain away but at least I held her hand and spoke to her as the nurses told me to.’ (Participant 50, male, birth companion of choice)

Other studies have also reported that male partners view childbirth as a transformative experience in their lives, which is marked by intense emotions and the need for confirmation in their supporting role (Backstrom & Herfelt Wahn 2011; Johansson, Fenwick & Premberg 2015).

### Compassion and affection of healthcare workers

A study in China revealed that showing concern, being kind and friendly and treating everyone equal are particularly important qualities that healthcare providers are expected to demonstrate. These attributes are much valued by women in labour (Raven et al. 2015). Similarly, the birth companions in the study specifically recalled, with appreciation, the compassion and affection shown by healthcare workers towards the women in labour, which underpins the entire experience of the care received:

‘I was very happy with the way the staff treated my sister. They are really quality staff and provided my sister with good tender care. I rate the staff 10/10!.’ (Participant 10, female, birth companion of choice)

‘They were patient with us, also very much friendly and kind.’ (Participant 29, female, birth companion of choice)

‘They were friendly to me and to my child. I appreciate that. Thanks for everything nurses.’ (Participant 43, female, birth companion of choice)

‘We have been welcomed with great love. The service was good from the start to the end. We thank you for the great work that you do, not just to us only but to other people who are coming here. Thank you so much.’ (Participant 30, female, birth companion of choice)

‘It was a joyous event. Though I was exhausted, I could still feel the great service that they offered us.’ (Participant 69, female, birth companion of choice)

Birth companions can also bear witness to disrespectful and abusive behaviour towards women in labour. Although their role is not to disguise the negative behaviour, they can play a very important role to buffer the impact of the disrespect and abuse during labour and birth, as well as provide crucial details of the encounter to ensure accountability and eradication of disrespectful behaviour.

One birth companion who had a negative experience reported the following:

‘The nurse who was helping to deliver the baby was impatient and rude to the mother. Once the baby was delivered and she had to stitch the mother’s vagina where she had cut her, the mother felt a lot of pain and kept moving backwards on the bed. The nurse kept shouting at the mother for moving away and told her that she is going to dirty her [*nurse*] uniform whereas she [*nurse*] still has other places to go when she knocks off, unlike the mother. I gave the mother my hand to hold onto as she was being stitched and she almost dug holes into my hand. I felt so sad for the mother … it almost felt like I was the one feeling her pain. It really put a damper on the beautiful experience of the labour and birth.’ (Participant 4, female, volunteer birth companion)

One other birth companion witnessed disrespectful behaviour towards a woman who gave birth on the floor in the passage of the postnatal ward:

‘The nurse started screaming at the mother and blaming her for what was happening as if it was her fault that the baby started coming when she was in the passage. Then the nurse started screaming at everyone to go away … I understand she did not want everyone around watching the mother giving birth but she was very rude in how she was speaking to the mother and to everyone in the passage.’ (Participant 49, male, birth companion of choice)

## Discussion

The study aimed to enhance insights into the perceptions and experiences of birth companions of choice, as well as volunteer birth companions, about their role in providing continuous physical and emotional support to women during labour and birth. The study revealed that birth companions generally have positive experiences and perceptions about their role. Their experiences are directly influenced by the attitudes of healthcare workers towards the women that they support during labour and birth.

The RMC model that is implemented at the study hospital advocates for all women to be continuously supported by a birth companion during labour and birth. The model also advocates for birth companions to be orientated on their roles and responsibilities as birth companions upon arrival in the labour ward. The orientation appears to have contributed to the positive experience of birth companions, by increasing their sense of confidence and perceived value of their role. Providing physical and emotional support to women in labour is the core purpose and function of birth companions, and thus deriving satisfaction from fulfilling their role is a testament to the need for birth companions during labour and birth.

Thus, it is important to invest in equipping birth companions with the necessary knowledge and skills to execute their roles and responsibilities during labour and birth. A competent and confident birth companion not only enhances the experience of labour and birth for the woman being supported but also contributes to the acceptability of birth companions by healthcare providers.

Although male partners as birth companions have been associated with reported challenges that include cultural and practical considerations, it does not take away from the important role that they play in family-centred care during labour and birth. The males that participated in the study reported positive experiences of their role in supporting their partners. Far more effort should be made in debunking the myths and misperceptions about male partners as birth companions and normalising their role in supporting women during labour and birth.

One other key role of birth companions, which is not always appreciated by healthcare providers, is one of holding healthcare providers accountable. The birth companions are key witnesses to both positive and negative experiences in the labour ward. Their mere presence in the labour ward should, and often does, deter healthcare providers from providing disrespectful and abusive care to women in labour. Accountability is critical for improving the quality of care provided by a health facility, and therefore positive and negative feedback from birth companions should be incorporated into ongoing quality improvement strategies.

### Strengths and limitations

Although the study provides valuable insights for the integration of birth companions into the labour ward, there are a number of limitations that need to be taken into account. For one, there were no measures to enforce healthcare providers encouraging all birth companions to document their experiences in the birth companion feedback book. The onus was entirely on midwives attending to a birth to do so. Furthermore, it is also possible that some birth companions could have felt intimidated to provide negative feedback in the book, which is placed at the nurse’s station, in spite of the option for them to remain anonymous.

## Conclusion

The RMC project that was piloted advocates for women to be supported continuously by a birth companion of choice during labour and childbirth. Documenting the experiences and perceptions of birth companions who are supporting women is critical for the continuous improvement of the quality and experience of care by pregnant women during labour and childbirth. It is important for healthcare providers to understand the far reaching emotional and psychological impact of their attitudes and behaviour on not only the women in labour but also on others who witness their (healthcare providers) behaviour. Mechanisms to obtain feedback from birth companions should be integrated into the strategies to improve the quality and experience of care for women during labour and childbirth.
